# Prevalence of radiographic hip osteoarthritis is increased in high bone mass

**DOI:** 10.1016/j.joca.2014.06.007

**Published:** 2014-08

**Authors:** S.A. Hardcastle, P. Dieppe, C.L. Gregson, D. Hunter, G.E.R. Thomas, N.K. Arden, T.D. Spector, D.J. Hart, M.J. Laugharne, G.A. Clague, M.H. Edwards, E.M. Dennison, C. Cooper, M. Williams, G. Davey Smith, J.H. Tobias

**Affiliations:** † Musculoskeletal Research Unit, School of Clinical Sciences, University of Bristol, UK; ‡ MRC Integrative Epidemiology Unit, University of Bristol, UK; § University of Exeter Medical School, Exeter, UK; ‖ Oxford NIHR Musculoskeletal Biomedical Research Unit, University of Oxford, Oxford, UK; ¶ Chromatic Innovation Limited, 23 Chesham St, Leamington Spa, CV31 1JS, UK; # MRC Lifecourse Epidemiology Unit, University of Southampton, Southampton, UK; †† Arthritis Research UK (ARUK) Centre for Sports, Exercise and Osteoarthritis, University of Oxford, Nuffield Orthopaedic Centre, Oxford, UK; ‡‡ Department of Twin Research and Genetic Epidemiology, King's College London, London, UK; §§ Department of Radiology, Royal United Hospital Bath NHS Trust, Bath, UK; ‖‖ Department of Radiology, Royal Glamorgan Hospital, Cwm Taf Health Board, Llantrisant, Wales, UK; ¶¶ NIHR Nutrition Biomedical Research Centre, University of Southampton, Southampton, UK; ## Department of Radiology, North Bristol NHS Trust, Bristol, UK

**Keywords:** Osteoarthritis, Osteoporosis, DXA, Radiology, Epidemiology

## Abstract

**Objective:**

Epidemiological studies have shown an association between increased bone mineral density (BMD) and osteoarthritis (OA), but whether this represents cause or effect remains unclear. In this study, we used a novel approach to investigate this question, determining whether individuals with High Bone Mass (HBM) have a higher prevalence of radiographic hip OA compared with controls.

**Design:**

HBM cases came from the UK-based HBM study: HBM was defined by BMD Z-score. Unaffected relatives of index cases were recruited as family controls. Age-stratified random sampling was used to select further population controls from the Chingford and Hertfordshire cohort studies. Pelvic radiographs were pooled and assessed by a single observer blinded to case-control status. Analyses used logistic regression, adjusted for age, gender and body mass index (BMI).

**Results:**

530 HBM hips in 272 cases (mean age 62.9 years, 74% female) and 1702 control hips in 863 controls (mean age 64.8 years, 84% female) were analysed. The prevalence of radiographic OA, defined as Croft score ≥3, was higher in cases compared with controls (20.0% vs 13.6%), with adjusted odds ratio (OR) [95% CI] 1.52 [1.09, 2.11], *P* = 0.013. Osteophytes (OR 2.12 [1.61, 2.79], *P* < 0.001) and subchondral sclerosis (OR 2.78 [1.49, 5.18], *P* = 0.001) were more prevalent in cases. However, no difference in the prevalence of joint space narrowing (JSN) was seen (OR 0.97 [0.72, 1.33], *P* = 0.869).

**Conclusions:**

An increased prevalence of radiographic hip OA and osteophytosis was observed in HBM cases compared with controls, in keeping with a positive association between HBM and OA and suggesting that OA in HBM has a hypertrophic phenotype.

## Introduction

Epidemiological studies have identified increased bone mineral density (BMD) as a potential risk factor for osteoarthritis (OA). For example, cross-sectional studies have demonstrated associations between increased BMD and both radiographic hip^1^, ^2^ and knee^3^, ^4^ OA in a variety of populations, and longitudinal studies have associated increased BMD with incident knee^5^, ^6^, ^7^, ^8^ and hip^9^ OA. In addition, several studies have observed a stronger association between BMD and osteophytosis than that with joint space narrowing (JSN) (indicative of cartilage loss)^1^, ^3^, ^9^, suggesting that increased BMD predisposes primarily to the bony features of OA. However, while the epidemiological association between increased BMD and radiographic OA is generally accepted, the topic remains controversial as it is possible that confounding^10^ or reverse causality (in cross-sectional studies) may explain the relationships observed.

Studying a high bone mass (HBM) population represents a novel way to examine the OA-BMD relationship. As HBM is likely to be a lifelong genetically-determined trait, and OA is a disease of later life, this approach avoids uncertainty over the temporal sequence of events which complicates the interpretation of previous cross-sectional studies. Existing data on OA in association with extreme HBM phenotypes is limited to case reports and case series, and to our knowledge radiographic OA in a HBM population has never been systematically studied. We previously reported an increased prevalence of prior joint replacement, particularly hip replacement, in HBM cases within our UK-based HBM study compared with family controls^11^. While this suggests that OA risk may be elevated in HBM, joint replacement captures only end-stage disease, and provides limited information on OA phenotype. Recent pQCT analysis of this same HBM population revealed increases in total bone area in cases compared with controls suggestive of increased periosteal apposition^12^, implying increased bone formation. This raises interesting parallels with OA, as alterations in the balance between bone formation and resorption are suggested to be a key component of the disease^13^, ^14^.

The aim of our study was to quantify and characterise radiographic hip OA in a population of individuals with extreme HBM. We wished to determine (1) whether the prevalence of radiographic hip OA is increased in HBM compared with both family-based and general population controls and (2) whether the OA observed in HBM has a characteristic phenotype based upon individual radiographic features of the disease. We hypothesized that the prevalence of radiographic OA would be increased in HBM cases, and that HBM may be associated with an excess of bone-forming features such as osteophytes and subchondral sclerosis.

## Methods

### The HBM population

The HBM study is a UK-based multi-centre observational study of adults with unexplained HBM. 335,115 DXA scans from 13 UK DXA databases were screened for T and/or Z-scores ≥+4. All DXA images were inspected by trained clinicians for artefactual causes of elevated DXA BMD; 49.4% of scans were excluded as their high T-/Z-scores reflected spinal degenerative disease/osteoarthritis/scoliosis, and a further 15.5% for other reasons including surgical/malignant/Pagetic artefacts etc. As generalized HBM should affect both hip and spine BMD, the HBM index case definition was refined to either a) L1 Z-score ≥+3.2 plus total hip Z-score ≥+1.2 or b) total hip Z-score ≥+3.2 plus L1 Z-score ≥+1.2. While standard definitions of HBM are lacking, a +3.2 threshold was similar to that used in a previous publication defining HBM using DXA^15^, and most appropriately differentiated generalized HBM from artefact^16^. Misclassification of HBM case status due to lumbar OA was minimized by using L1 Z-score which, in contrast to lower lumbar levels, was not associated with OA assessed on DXA images^16^, ^17^.

Recruited index cases with unexplained HBM were asked to invite relatives and spouses to undergo DXA screening. In first-degree relatives of HBM index cases, given positive affection status within the family, HBM was defined as a summed L1 Z-score plus total hip Z-score ≥+3.2. 41% of relatives screened were affected and combined with HBM index cases; remaining unaffected first-degree relatives/spouses formed a family control group. Full details of this DXA database screening and recruitment were previously reported^16^. Assessments in both HBM cases and controls included a structured interview and clinical examination. Supine AP pelvic radiographs were performed in participants aged ≥40 years according to local protocols at each centre. Recruitment ran from July 2005–April 2010. Written informed consent was obtained from all participants in line with the Declaration of Helsinki^18^ and the study was approved by the Bath multi-centre Research Ethics Committee (REC) and each NHS local REC. For this study, HBM cases were then categorised into 5-year age bands by gender, prior to selection of additional controls from two large population-based cohort studies, by age and gender-stratified random sampling.

### Population-based controls

#### Chingford 1000 women study controls

The Chingford 1000 women study (ChS) started in 1989, initially recruiting 1003 women aged 45–64 from the age/sex register of a general practice in Chingford, North-East London^4^. 470 women (46.9%) remained under radiographic follow-up at 20 years. Supine pelvic radiographs were obtained in years 2, 8 and 20; radiographs from year 20 were digital and those from years 2 and 8 latterly digitised. Controls, according to age at the time of X-ray, were randomly sampled in a 2:1 ratio with HBM female cases for each age band apart from the lower (40–50 years) and upper (>80) bands (3:1). Where a control individual had more than one pelvic radiograph from different follow-up time-points, a single radiograph per participant was included; controls in the upper age bands were selected first to ensure sufficient numbers of available X-rays.

#### Hertfordshire cohort study (HCS) controls

The HCS^19^ recruited approximately 3000 men and women born in Hertfordshire between 1931 and 1939 and still resident there in 1998–2003. Recently a subset of HCS participants were recruited into the European Project on Osteoarthritis (EPOSA)^20^, as part of which 207 men and 203 women now aged between 71.5 years and 80.6 years had AP weight-bearing knee and/or supine pelvic X-rays performed during 2011. These individuals were randomly sampled 2:1 with HBM cases within each appropriate age band (70–75, 75–80 and >80).

### Assessment of radiographs

All available case and control radiographs were pooled for assessment; reasons for unavailability of individual X-rays were ascertained and recorded. Radiographs were blinded and graded in a random order by a single observer (SH), following focussed radiological training. Radiographic assessment was performed using OxMorf v1.6,^1^ a bone morphology measurement system developed by the University of Oxford^21^. The software was used to record gradings of the radiographic OA features, and to measure minimum joint space width (JSW) quantitatively. However, as differences in radiographic protocols between studies can result in varying degrees of magnification of the X-ray image, we could not reliably compare quantitative measures between studies; analysis of measured JSW was therefore limited to the HBM cases and family controls only.

Radiographs were first given an overall OA grade using the Croft score (0–5) as originally defined^22^, followed by semi-quantitative grading of individual radiographic features including osteophytes, JSN, cysts and sclerosis (Table I) using an established atlas^23^. Categorical scores were converted to binary variables for analysis (Table I); a Croft grade of ≥3 (defined as two of osteophytosis, JSN, subchondral sclerosis or cyst formation^22^) defined the presence of OA^24^. Measurement of minimum JSW involved manual placement of two Bézier curves on the acetabular rim and femoral head by the operator– the OxMorf software then calculated the minimum distance between the two lines.

**Table I:** Semi-quantitative scoring of radiographic features of hip OA. Grading of individual radiographic features (except chondrocalcinosis) was performed using an atlas^23^. Croft grading performed with reference to original published descriptions of each grade^22^. The assessor was permitted to enlarge each image as necessary to visualise the features. OP = osteophyte

OA feature	Categorical grading	Binary variable (s)
Croft score (global hip OA)	0–5	Croft score ≥3 (OA present)
Superior acetabular osteophyte	0–3	Any osteophyte (any OP grade ≥1), moderate osteophyte (any OP grade ≥2), femoral osteophyte (any femoral OP grade ≥1)
Lateral femoral osteophyte	0–3	
Medial femoral osteophyte	0–3	
JSN	0–3	Any JSN (JSN grade ≥1), moderate JSN (JSN grade ≥2)
Subchondral sclerosis	0–1	Subchondral sclerosis (grade ≥1)
Cysts	0–1	Cysts (grade ≥1)
Chondrocalcinosis	0–1	Chondrocalcinosis (grade ≥1)

Image quality was rated by the operator at the time of assessment (good, poor, very poor); very poor films in terms of penetration/resolution/tilt/rotation were excluded. The presence of joint replacements was recorded and these hips excluded from the main analysis (a later sensitivity analysis included these films). At the end of the study, 5% of radiographs (*n* = 60 films, 119 hips [1 hip replacement]) were re-graded, blind, to establish intra-rater repeatability; kappa values were all >0.7 except for cysts (kappa 0.32, likely reflecting the very low prevalence of cysts overall) (Supplementary Table 1). The intraclass correlation coefficient (ICC) for minimum measured JSW was 0.89.

### Assessment of covariates

Values for age, gender and body mass index (BMI) were obtained from each pre-existing study dataset for use in the analysis. Age was the age in years at the time of X-ray. BMI was calculated as weight (kg)/height (metres^2^) using the closest available weight and height measurements to the time of the X-ray.

### Statistical analysis

Demographic statistics for the different study populations were summarised as mean (SD) for continuous variables and counts (percentages) for categorical variables. In this case–control analysis, categorical variables were initially cross-tabulated and percentages calculated. The chi-squared (χ^2^) test was used to assess the association between binary variables, and the unpaired *t*-test to compare mean values for continuous JSW. Generalised estimating equations (GEE) using a logistic link function (logistic regression allowing for clustering of observations within individuals, i.e. right/left hips) were used to examine associations between HBM case status and the radiographic OA outcome variables. Age and gender were *a priori* confounders and BMI was an additional potential confounder. Odds ratios (OR) before (model 1) and after (model 2) adjustment are presented with 95% confidence intervals (95% CI). GEE using an identity link function (linear regression allowing for clustering) was used to compare minimum JSW (mm) in HBM cases and family controls, adjusting for confounders. Pre-planned sensitivity analyses included (1) exclusion of films rated as poor quality (2) a “person-level” analysis of the worst hip in each individual (3) adding radiographic hip replacements to the dataset, assuming these were performed for OA (4) excluding individuals with self-reported inflammatory arthritis and (5) re-defining hip OA using different Croft grade cut-offs. Interactions by age and gender were assessed by calculating OR according to categories of each variable, and generating a wald test *P*-value for appropriate interaction terms. Data were analysed using Stata release 12 statistical software (StataCorp, College Station, TX, USA).

## Results

### Selection and characteristics of participants

**Fig. 1:**
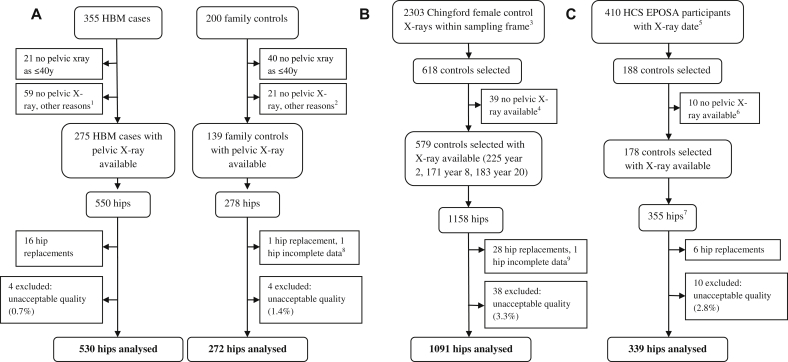
(A) Selection of HBM case and family control X-rays (process of recruitment to study previously reported^16^). (B) Selection of Chingford study female control X-rays. (C) Selection of HCS EPOSA male and female control X-rays. ^1^Reason recorded for missing X-ray in HBM cases: unable to travel (*n* = 7), no X-rays at study centre (*n* = 23), unable to attend/wait/comply (*n* = 4), patient declined (*n* = 8), not done (reason unknown) (*n* = 9), reside abroad (*n* = 2), bilateral hip replacements (*n* = 6). ^2^Reason recorded for missing X-ray in family controls: did not continue in study (*n* = 1), unable to travel (*n* = 1), no X-rays at study centre (*n* = 9), unable to attend/wait/comply (*n* = 2), patient declined (*n* = 4), not done (reason unknown) (*n* = 3), bilateral hip replacements (*n* = 1). ^3^Sampling frame constructed from dates of year 2, 8 and 20 follow-up visits supplied by study team. ^4^Reason recorded for missing X-ray in Chingford controls: not found at time of request (*n* = 6), not digitised (*n* = 18), unknown reason (*n* = 15). ^5^Sampling frame constructed from study X-ray appointment dates supplied by study team. ^6^Reason recorded for missing X-ray in HCS controls: bilateral hip replacements (*n* = 3), unknown (*n* = 7). ^7^One individual contributed only one hip. ^8^Excluded as missing lateral femoral osteophyte variable. ^9^Excluded as previous fracture with fixation device *in situ* precluding reliable assessment.

Figure 1 summarises the selection of radiographs for inclusion in our study. 56 hip joints (*n* = 4 cases, 52 controls) were excluded from the outset due to unacceptable image quality. Hip replacements were also excluded (*n* = 16 cases, 35 controls). 2232 hips from 1135 individuals were included in the primary combined analysis comprising 530 HBM case hips, 272 family control hips, 1091 ChS control hips and 339 HCS control hips. 1097 individuals contributed two hips to the analysis and 38 individuals contributed only one hip. Demographics of the study population are shown in Table II. The combined control group was slightly older than the cases (mean age 64.8 years vs 63.0 years) with a higher proportion of females (84.4% vs 74.3%). As expected, cases had substantially higher values for standardised BMD at both hip and lumbar spine compared with controls. Cases also had a higher mean BMI (30.5 kg/m^2^ vs 27.3 kg/m^2^), as previously reported^16^.

**Table II:** Demographics of study population

	HBM cases (*N* = 272)	Family controls (*N* = 137)	Chingford controls (*N* = 553)	HCS controls (*N* = 173)	Combined controls (*N* = 863)
	Mean (SD)	Mean (SD)	Mean (SD)	Mean (SD)	Mean (SD)
Age (years)	63.0 (11.5)	59.9 (12.7)	62.8 (9.8)	75.2 (2.7)	64.8 (10.8)
BMI (kg/m^2^)	30.5 (5.7)	28.1 (4.9)	27.0 (4.6)	27.7 (4.4)	27.3 (4.7)
BMD total hip (g/cm^2^)	1.25 (0.17)∗	0.98 (0.14)‡	0.90 (0.12)§	0.96 (0.14)	0.93 (0.13)¶
BMD L1–L4 (g/cm^2^)	1.55 (0.18)†	1.17 (0.18)‡	1.03 (0.16)‖	1.09 (0.19)	1.07 (0.18)#
	***N* (%)**	***N* (%)**	***N* (%)**	***N* (%)**	***N* (%)**
Females	202 (74.3)	61 (44.5)	553 (100.0)	114 (65.9)	728 (84.4)

BMD variables standardised according to scanner type (Hologic for Chingford/HCS controls, mixed Lunar/Hologic for HBM cases and family controls) using standard equations^46^, ^47^.

*N* for all variables is as shown except where indicated:

∗ *N* = 264

† *N* = 261

‡ *N* = 136

§ *N* = 479

‖ *N* = 539

¶ *N* = 788

# *N* = 848

### Radiographic OA in HBM cases vs controls: unadjusted analyses

The unadjusted prevalence of each radiographic OA variable in cases was compared with that in each of the three control groups separately and then combined (Table III). The prevalence of radiographic hip OA (defined as Croft score ≥3) was 20.0% in HBM cases and 13.6% in the combined controls (*P* < 0.001). The prevalence of any osteophyte, moderate (≥grade 2) osteophytes, femoral osteophytes, subchondral sclerosis and chondrocalcinosis was also greater in cases compared with combined controls. No difference was observed between groups in the prevalence of JSN or cysts.

**Table III:** Prevalence of hip OA features in HBM cases and control groups

	HBM cases (*N* = 530)	Family controls (*N* = 272)		Chingford controls (*N* = 1091)		HCS controls (*N* = 339)		Combined controls (*N* = 1702)	
	*N* (%)	*N* (%)	χ^2^*P*	*N* (%)	χ^2^*P*	*N* (%)	χ^2^*P*	*N* (%)	χ^2^*P*
Croft score ≥3	106 (20.0)	46 (16.9)	0.291	102 (9.4)	<0.001	83 (24.5)	0.118	231 (13.6)	<0.001
Osteophyte (any)	373 (70.4)	166 (61.0)	0.008	546 (50.5)	<0.001	225 (66.4)	0.214	937 (55.1)	<0.001
Moderate (≥grade 2) osteophyte	117 (22.1)	32 (11.8)	<0.001	97 (8.9)	<0.001	52 (15.3)	0.014	181 (10.6)	<0.001
Any femoral osteophyte	108 (20.4)	36 (13.2)	0.013	115 (10.5)	<0.001	76 (22.4)	0.472	227 (13.3)	<0.001
JSN (any)	116 (21.9)	55 (20.2)	0.585	173 (15.9)	0.003	125 (36.9)	<0.001	353 (20.7)	0.572
Moderate (≥grade 2) JSN	22 (4.2)	8 (2.9)	0.393	20 (1.8)	0.006	22 (6.5)	0.125	50 (2.9)	0.167
Cysts	2 (0.4)	2 (0.7)	0.496	14 (1.3)	0.084	5 (1.5)	0.077	21 (1.2)	0.088
Sclerosis	30 (5.7)	7 (2.6)	0.049	16 (1.5)	<0.001	13 (3.8)	0.226	36 (2.1)	<0.001
Chondrocalcinosis	24 (4.5)	7 (2.6)	0.174	27 (2.5)	0.026	12 (3.5)	0.476	46 (2.7)	0.035
	**Mean (SD)**	**Mean (SD)**	***P***†	**Mean (SD)**	***P***	**Mean (SD)**	***P***	**Mean (SD)**	***P***
Measured JSW (mm)∗	3.05 (0.71)	3.06 (0.68)	0.754	–	–	–	–	–	–

*P* values refer to comparison with HBM cases. *N* for all variables is as shown except where indicated and refers to number of hip joints analysed.

∗ Quantitative measurement of JSW limited to HBM study participants (HBM cases and family controls) only; *N* = 526 (HBM cases), 270 (family controls).

† *P* value from unpaired *t*-test.

### Radiographic OA in HBM cases vs controls: adjusted analyses

As additional adjustment for BMI gave very similar results to age and gender adjustment alone, all three covariates were included in our fully adjusted model. Radiographic hip OA remained more prevalent in HBM cases compared with combined controls after full adjustment (OR [95% CI] 1.52 [1.09, 2.11], *P* = 0.013, model 2, Table IV). In adjusted analyses of individual radiographic features of OA, HBM cases had an increased odds compared with controls of any osteophyte (2.12 [1.61, 2.79] *P* < 0.001), moderate osteophytes (2.39 [1.72, 3.33], *P* < 0.001) and femoral osteophytes (1.60 [1.18, 2.17], *P* = 0.003). Other radiographic features more prevalent in cases compared with controls included subchondral sclerosis (2.78 [1.49, 5.18], *P* = 0.001) and chondrocalcinosis (2.08 [1.07, 4.03], *P* = 0.030) although the prevalence of these features was much lower than that of osteophytes and overall OA. In contrast, the prevalence of JSN was similar in cases and controls (0.97 [0.72, 1.33], *P* = 0.869), and there was no strong evidence of a difference between groups in the prevalence of subchondral cysts (0.34 [0.08, 1.42], *P* = 0.139).

**Table IV:** GEE regression analysis of radiographic hip OA variables in HBM cases vs all combined controls. Results show OR, with 95% confidence interval (95% CI). *N* (total no. of hip joints analysed) = 530 (HBM cases), 1702 (controls). Model 1 = unadjusted, model 2 = adjusted for age, gender and BMI. GEE = Generalised estimating equations with logistic link function

Outcome	Model	OR (95% CI) in HBM cases vs controls	*P*
Croft score ≥3	1	1.58 (1.17, 2.14)	0.003
	2	1.52 (1.09, 2.11)	0.013
Osteophyte (any)	1	1.94 (1.51, 2.49)	<0.001
	2	2.12 (1.61, 2.79)	<0.001
Moderate (≥grade 2) osteophyte	1	2.35 (1.72, 3.21)	<0.001
	2	2.39 (1.72, 3.33)	<0.001
Any femoral osteophyte	1	1.66 (1.23, 2.23)	0.001
	2	1.60 (1.18, 2.17)	0.003
JSN (any)	1	1.08 (0.81, 1.43)	0.598
	2	0.97 (0.72, 1.33)	0.869
Moderate (≥grade 2) JSN	1	1.44 (0.82, 2.53)	0.206
	2	1.48 (0.82, 2.69)	0.196
Cysts	1	0.30 (0.07, 1.28)	0.104
	2	0.34 (0.08, 1.42)	0.139
Sclerosis	1	2.81 (1.54, 5.13)	0.001
	2	2.78 (1.49, 5.18)	0.001
Chondrocalcinosis	1	1.62 (0.86, 3.05)	0.135
	2	2.08 (1.07, 4.03)	0.030

Separate analyses were performed comparing the prevalence of radiographic OA in HBM cases with that in (1) family controls (Supplementary Table 2) (2) ChS controls (females only, Supplementary Table 3) (3) HCS controls (Supplementary Table 4), and (4) between HBM male cases and all male controls (Supplementary Table 5). Findings were broadly consistent across these different subgroups, although confidence intervals were generally widened. The fully adjusted OR for hip OA was lower for HBM cases vs family controls (1.32 [0.77, 2.27], *P* = 0.318) than for HBM cases vs all pooled controls, however osteophytes remained strongly associated with HBM case status and the OR for subchondral sclerosis was similar to that seen overall. Minimum measured JSW did not differ between HBM cases and family controls (Supplementary Table 2). Restricting analyses to those HBM individuals aged ≥65 years vs HCS controls (due to the older age range of the HCS population, Supplementary Table 4) resulted in small numbers; whilst the OR for hip OA was attenuated (1.14 [0.70, 1.86], *P* = 0.608), a strong association persisted for the osteophyte variables.

### Interaction with age and gender

**Fig. 2:**
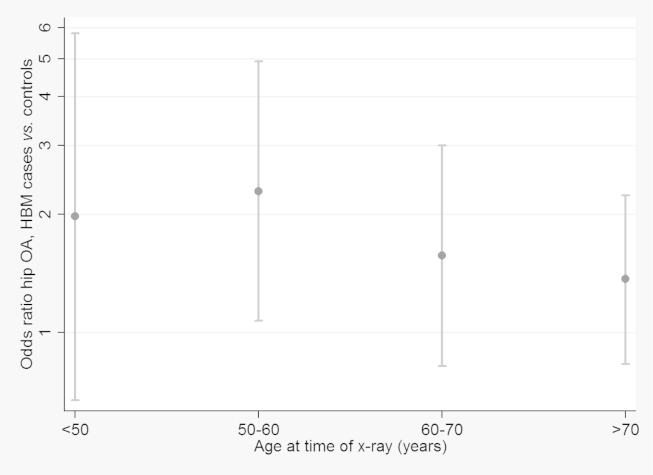
Figure shows OR for hip OA (defined as Croft score ≥3) in HBM cases vs controls, according to age group, adjusted for gender and BMI. <50 y *n* (no. of hips) = 324, 81.5% female; 50–60 y *n* = 420, 89% female; 60–70 y *n* = 669, 87.4% female; >70 y *n* = 819, 74% female. GEE used to account for within participant clustering (left/right).

The suggestion that the association of radiographic OA with HBM is attenuated in older individuals was explored by examining age interactions in analyses comparing HBM cases with combined controls. Some evidence of a HBM-age interaction was found, with the odds of OA in cases vs controls greater in the younger age groups (Fig. 2; wald test *P* value for interaction term 0.04), suggesting that HBM cases may develop OA at an earlier age. Whilst the overall prevalence of hip OA was greater in males compared with females (27.5% and 12.4% respectively), there was no evidence of a gender interaction (*P* = 0.59).

### Sensitivity analyses

31 case hips (5.7%) and 229 control hips (13.5%) were of poor quality in terms of resolution/penetration/artefact. Excluding these films resulted in point estimates that were essentially unchanged (OR for hip OA 1.57 [1.10, 2.23], *P* = 0.012; any osteophyte 2.14 [1.59, 2.88], *P* < 0.001; subchondral sclerosis 2.94 [1.52, 5.67], *P* = 0.001) (model 2). A person-level analysis of the worst hip per participant, also resulted in very similar point estimates (adjusted OR for hip OA 1.61 [1.14, 2.26, *P* = 0.007; any osteophyte 2.79 [1.91, 4.06], *P* < 0.001; subchondral sclerosis 2.40 [1.30, 4.43], *P* = 0.005). Although hip replacements were excluded from the main analysis, a sensitivity analysis was performed in which these hips were included, with total hip replacements (*n* = 50) assumed to have OA. This resulted in a small increase in the OR for hip OA in HBM (1.58 [1.15, 2.17], *P* = 0.005, model 2, Supplementary Table 6). For HBM study cases and controls only, data on self-reported inflammatory arthritis were available. Excluding the hips of these individuals from the analysis (*N* = 35 HBM cases, two family controls) resulted in only minor attenuation of the OR for hip OA overall (Supplementary Table 7), whilst a strong association with osteophytosis persisted; numbers for this analysis were comparatively small.

The effect of applying different Croft grade cut-offs for radiographic hip OA is shown in Supplementary Table 8; if the Croft grade 3 definition was modified to require the presence of JSN (≥grade 1), there was little evidence of attenuation (OR 1.42 [1.02, 1.98], *P* = 0.038). Defining radiographic hip OA as Croft grade ≥4 (“severe radiographic OA”^25^) strengthened the association with HBM (OR 1.99 [1.04, 3.81], *P* = 0.037), whereas a definition based on Croft grade ≥2^26^ resulted in no association between HBM and hip OA (OR 1.04 [0.76, 1.41], *P* = 0.802).

## Discussion

Our data are the first to support an increased prevalence of radiographic hip OA in a population of extreme HBM cases, compared with controls. Furthermore, features of OA reflecting excess bone formation (osteophytes and subchondral sclerosis) were more strongly associated with HBM case status than other OA features such as JSN and cysts, for which no clear association was seen. Taken with results from our recent pQCT study^12^, these findings suggest that increased bone formation is a key feature of the HBM phenotype, which in turn leads to a greater risk of OA. HBM was suggested to represent an incidental finding when originally described^16^, but our present results fit with more recent evidence that it is associated with significant co-morbidities^11^, ^27^. Our findings were generally consistent throughout the different control groups, other than HBM cases being more similar to family controls than to general population controls (a finding likely to be explained by shared genetic and environmental factors). Associations between HBM and radiographic features of hip OA persisted following adjustment for the confounders age, gender and BMI.

The extent to which findings in this extreme HBM group can be generalised to populations with a more typical BMD distribution is uncertain. Our observation that OA in HBM appeared to be primarily characterised by osteophytes rather than JSN likely reflects relationships between OA and BMD in the wider population, as osteophytes were reportedly more strongly associated with increased BMD than JSN in previous population-based studies^1^, ^3^, ^9^. JSN is considered the best radiographic surrogate of cartilage loss^28^, and is arguably a prerequisite for the definition of hip OA^29^. Our failure to find evidence of differences in JSN between groups might reflect methodological limitations. Assessment of JSN on radiographs is insensitive to early cartilage changes in comparison to MRI-based and arthroscopic methods^30^, ^31^, and while measurement of minimum JSW may have been more sensitive than semi-quantitative JSN assessment, unfortunately we were only able to compare this outcome in HBM cases and family controls.

Alternatively, it is possible that HBM predisposes primarily to the bony features of OA and not to cartilage loss. A distinction has been made in the literature between the relationship of increased BMD with incident knee OA (increased) and progression of existing knee OA (possibly reduced)^5^, ^6^ and, interestingly, a recent study reported a positive association between systemic BMD and MRI-measured cartilage thickness in radiographic knee OA, possibly indicating a protective role for BMD against cartilage degradation in established disease^32^. Similarly, although osteophytes and JSN are generally associated, not all joints with osteophytes progress over time to develop the other structural changes of OA^33^, ^34^. Osteophytes may even have a positive role in stabilising the OA joint against further deterioration^35^, possibly explaining why “atrophic” hip OA lacking osteophytes may progress more rapidly than hypertrophic forms of the disease^36^, ^37^. Against this hypothesis is our previous finding of increased joint replacement in HBM cases^11^ supporting an increase in clinically significant OA in HBM, although it is worth noting that bony changes of OA may themselves be an important source of pain^38^, ^39^ and furthermore that more severe radiographic appearances may affect the decision to offer joint replacement surgery^40^.

How radiographic hip OA should be defined remains a matter of debate and, as we expected, the OA definition used affected the strength of association with HBM that we observed. A more stringent Croft grade cut-off of ≥4 strengthened the association between HBM and OA. In contrast, a Croft grade cut-off of ≥2 (allowing OA to be defined by JSN alone) resulted in no association; this definition would include atrophic OA^29^, ^41^, which might potentially have distinct risk factors. Indeed it was recently reported in a large population-based cohort that atrophic hip OA may actually be associated with lower BMD^42^. Defining hip OA as a Croft grade ≥3 but with JSN as one of the two required features, is closest to the definition of “composite OA” proposed by Javaid^41^, since the second feature in the vast majority of cases will be osteophytes. Definitions of hypertrophic OA have focussed on osteophytes, particularly femoral osteophytes^29^, ^41^, however whereas some authors have defined hypertrophic OA as osteophytosis in the absence of JSN^29^, ^41^, other definitions have required both osteophytes and JSN to be present^43^. In our study, defining hypertrophic OA as femoral osteophytosis without JSN strengthened the HBM-OA association (OR 1.69 [1.18, 2.43], *P* = 0.005). Therefore HBM appears to be associated with both composite and hypertrophic, but not atrophic, hip OA phenotypes.

As outlined in our introduction, extreme HBM is likely to be genetically determined with onset of elevated bone mass relatively early in life; the genetic basis of increased BMD in our HBM population is currently being investigated. Therefore, despite the cross-sectional nature of this analysis, we assume the relationships that we found reflect either a causal pathway between higher BMD and increased risk of OA, or genetic pleiotropy. However, it remains theoretically possible that local features of OA at the hip and/or lumbar spine, such as osteophytes, subchondral sclerosis and buttressing (in which osteophytes at the hip joint extend across the femoral neck), could have led to artefactual elevation of DXA BMD resulting in misclassification of case status. If this were the case, a spurious HBM-OA association could be induced. Every effort was made within the study design to avoid this; DXA scans were visually inspected for artefactual causes of raised BMD including significant OA, and the L1 vertebra was selected for the case definition as L1 Z-score was not associated with features of OA visible on lumbar DXA^16^, ^17^. In fact, this approach may have led to some individuals with both HBM and OA being excluded, which if anything would bias the results of the present study towards the null. Reassuringly, limited evidence from the literature suggests that hip OA has only a minimal influence on measured hip BMD^44^, and no association was observed between hip OA and ipsilateral total hip Z-score when HBM study participants were stratified by case status implying that hips affected and unaffected by OA had similar degrees of BMD elevation (Supplementary Table 9).

The fact that HBM cases were identified from a DXA population whereas controls came from other sources is a limitation of this study; whilst both ChS and HCS are thought to be broadly representative of the UK population^4^, ^19^, we cannot exclude the possibility that OA risk in a population referred for DXA scanning may be different. In addition, some radiographs for both cases and controls were unavailable (Fig. 1). Less mobile participants with OA may have been less likely to have attended for X-rays, and there was evidence in the HBM and HCS groups that hip replacements were less likely to be X-rayed, although numbers missing for this reason were small. In addition, our sampling strategy for the ChS controls (later X-rays selected first) was unavoidably biased towards those women with continued follow-up, possibly resulting in a “healthy cohort” effect. Both of these factors would have led to underestimation of the true OA prevalence amongst the population controls. However, as only 41% of HBM cases initially identified from DXA database screening were recruited into the HBM study^16^, participation bias in the same direction is likely to have affected the HBM group. A single observer graded all radiographs, which may have led to either over or underestimation of the prevalence of OA but should not have affected between-group differences; this strategy was chosen as intra-rater repeatability of semi-quantitative OA scoring is known to be substantially superior to that between observers^45^. Although every effort was made to blind the assessor to case–control status, complete blinding may have been lost for some older, poorer quality control images. Lastly, this study did not attempt to examine associations between radiographic OA and clinical features such as pain (however we have previously reported a comparison of clinical OA phenotypes including joint pain in HBM cases vs family controls^11^).

In conclusion, we have observed an increased prevalence of radiographic hip OA in HBM cases compared with both family-based and general population controls. In addition, those features of OA arising from increased bone formation, including osteophytes and subchondral sclerosis, were particularly strongly associated with HBM suggesting that hip OA in this group has a hypertrophic phenotype. These data support a true association between increased BMD and OA, and suggest that common underlying mechanisms relating to bone mass regulation may underpin both HBM and hypertrophic OA.

## Author contributions

Study design: SAH, PD, CLG, JHT, GDS, NKA, TDS, DJH, ED, CC. Study conduct: SAH, CLG, PD, MW, JHT, ME, ED, CC, TDS, DJH, DH, GT, NKA. Data collection: SAH, PD, CLG, GAC, MJL, ME, MW. Data analysis: SAH, CLG. Data interpretation: SAH, CLG, PD, GDS, JHT, NKA. Drafting manuscript: SAH, CLG, PD, JHT. Revising manuscript content: all authors. Approval of final draft: all authors. SAH takes responsibility for the integrity of the data analysis.

## Role of the funding source

This work was supported by was supported by the Wellcome Trust and the NIHR CRN (portfolio number 5163) (study design and recruitment). CLG was funded through a Wellcome Trust Clinical Research Training Fellowship (080280/Z/06/Z). Ongoing support is being provided by 10.13039/501100000341Arthritis Research UK, who also fund SH through a Clinical PhD Studentship (grant ref 19580) and CLG through a Clinician Scientist Fellowship (grant ref 20000). The Hertfordshire cohort study is supported by the 10.13039/501100000265MRC, Arthritis Research UK and the NIHR Nutrition Biomedical Research Centre, University of Southampton. Funders had no role in the design, conduct or analysis of the present study.

## Competing interest statement

The authors declare no competing interests relevant to this work.

## Footnotes

1 Previously known as “HipMorf”.
