# Supplementary material for: Prevalence of radiographic hip osteoarthritis is increased in high bone mass
Source: Osteoarthritis Cartilage. 2014 Aug;22(8):1120–8. doi: 10.1016/j.joca.2014.06.007 (PMC4147962; doi:10.1016/j.joca.2014.06.007)
Supplement: Supplementary file 1 [file mmc1.docx]

| **Outcome** | **Kappa (95% CI)** |
| --- | --- |
| **Croft score (cutoff ≥3)** | 0.78 (0.64, 0.93) |
| **Any osteophyte (≥grade 1)** | 0.73 (0.60, 0.86) |
| **Moderate osteophyte (≥grade 2)** | 0.74 (0.58, 0.90) |
| **Any femoral osteophyte (≥grade 1)** | 0.71 (0.55, 0.87) |
| **JSN (≥grade 1)** | 0.82 (0.70, 0.94) |
| **Moderate JSN (≥grade 2)** | 0.90 (0.72, 1.00) |
| **Cysts** | 0.32 (-0.17, 0.81) |
| **Sclerosis** | 0.80 (0.41, 1.00) |
| **Chondrocalcinosis** | 0.90 (0.72, 1.00) |

**Supplementary table 1**

**Intra-rater repeatability scores for the different binary radiographic features.** Generated by repeat blinded grading of 60 randomly selected X-rays (119 hips) by the primary observer, a minimum of 2 weeks after initial grading. Unweighted kappa values shown. Confidence intervals calculated using the ‘kapci’ command in Stata.

**Supplementary table 2**

| **Outcome** | **Model** | **OR (95% CI) in HBM cases *vs.* controls** | **p** |
| --- | --- | --- | --- |
| **Croft score ≥3** | 1 | 1.24 (0.77, 1.99) | 0.372 |
|  | 2 | 1.32 (0.77, 2.27) | 0.318 |
| **Osteophyte (any)** | 1 | 1.52 (1.04, 2.23) | 0.031 |
|  | 2 | 1.72 (1.13, 2.61) | 0.011 |
| **Moderate (≥grade 2) osteophyte** | 1 | 2.16 (1.27, 3.65) | 0.004 |
|  | 2 | 2.05 (1.14, 3.69) | 0.017 |
| **Any femoral osteophyte** | 1 | 1.70 (1.03, 2.81) | 0.038 |
|  | 2 | 1.98 (1.14, 3.43) | 0.015 |
| **JSN (any)** | 1 | 1.13 (0.72, 1.75) | 0.599 |
|  | 2 | 1.12 (0.68, 1.84) | 0.659 |
| **Moderate (≥grade 2) JSN** | 1 | 1.44 (0.62, 3.35) | 0.399 |
|  | 2 | 1.52 (0.58, 3.96) | 0.391 |
| **Cysts** | 1 | 0.51 (0.07, 3.64) | 0.503 |
|  | 2 | 0.54 (0.04, 7.39) | 0.642 |
| **Sclerosis** | 1 | 2.34 (0.82, 6.65) | 0.111 |
|  | 2 | 3.26 (0.92, 11.6) | 0.067 |
| **Chondrocalcinosis** | 1 | 1.65 (0.61, 4.46) | 0.327 |
|  | 2 | 1.66 (0.58, 4.79) | 0.347 |
|  | **Model** | **Mean difference (95% CI)** | **p** |
| **Measured JSW (mm)^1^** | 1 | -0.02 (-0.15, 0.11) | 0.755 |
|  | 2 | 0.09 (-0.05, 0.22) | 0.204 |

**GEE regression analysis of radiographic hip OA variables in HBM cases *vs.* family controls.** N (total no. of hip joints analysed) = 530 (HBM cases), 272 (family controls) except where indicated. Model 1=unadjusted, model 2= adjusted for age, gender and BMI. GEE = Generalised estimating equations with logistic / identity link function. ^1^N=526 (HBM cases), 270 (controls).

**Supplementary table 3**

| **Outcome** | **Model** | **OR (95% CI) in HBM cases *vs*. controls** | **p** |
| --- | --- | --- | --- |
| **Croft score ≥3** | 1 | 1.71 (1.14, 2.56) | 0.010 |
|  | 2 | 1.72 (1.11, 2.68) | 0.015 |
| **Osteophyte (any)** | 1 | 2.00 (1.51, 2.66) | <0.001 |
|  | 2 | 2.23 (1.63, 3.05) | <0.001 |
| **Moderate (≥grade 2) osteophyte** | 1 | 2.43 (1.63, 3.60) | <0.001 |
|  | 2 | 2.36 (1.54, 3.60) | <0.001 |
| **Any femoral osteophyte** | 1 | 1.65 (1.13, 2.42) | 0.010 |
|  | 2 | 1.63 (1.11, 2.41) | 0.013 |
| **JSN (any)** | 1 | 1.17 (0.81, 1.70) | 0.405 |
|  | 2 | 1.04 (0.69, 1.56) | 0.853 |
| **Moderate (≥grade 2) JSN** | 1 | 1.66 (0.74, 3.71) | 0.217 |
|  | 2 | 1.86 (0.76, 4.53) | 0.174 |
| **Cysts** | 1 | 0.19 (0.02, 1.46) | 0.111 |
|  | 2 | 0.21 (0.02, 1.76) | 0.149 |
| **Sclerosis** | 1 | 2.36 (0.96, 5.84) | 0.062 |
|  | 2 | 2.93 (1.09, 7.88) | 0.034 |
| **Chondrocalcinosis** | 1 | 1.16 (0.48, 2.78) | 0.741 |
|  | 2 | 1.59 (0.65, 3.92) | 0.313 |

**GEE regression analysis of radiographic hip OA variables in HBM female cases *vs.* Chingford Study controls.** N (total no. of hip joints analysed) = 394 (HBM cases), 1091 (Chingford controls) except where indicated. Model 1=unadjusted, model 2=adjusted for age and BMI. GEE = Generalised estimating equations with logistic link function.

**Supplementary table 4**

| **Outcome** | **Model** | **OR (95% CI) in HBM cases *vs*. controls** | **p** |
| --- | --- | --- | --- |
| **Croft score ≥3** | 1 | 1.19 (0.75, 1.89) | 0.452 |
|  | 2 | 1.14 (0.70, 1.86) | 0.608 |
| **Osteophyte (any)** | 1 | 1.79 (1.13, 2.84) | 0.013 |
|  | 2 | 2.00 (1.19, 3.34) | 0.008 |
| **Moderate (≥grade 2) osteophyte** | 1 | 2.38 (1.44, 3.93) | 0.001 |
|  | 2 | 2.34 (1.38, 3.97) | 0.002 |
| **Any femoral osteophyte** | 1 | 1.03 (0.64, 1.67) | 0.899 |
|  | 2 | 1.04 (0.63, 1.73) | 0.866 |
| **JSN (any)** | 1 | 0.72 (0.46, 1.11) | 0.140 |
|  | 2 | 0.66 (0.42, 1.05) | 0.078 |
| **Moderate (≥grade 2) JSN** | 1 | 0.89 (0.39, 2.02) | 0.781 |
|  | 2 | 0.92 (0.40, 2.10) | 0.837 |
| **Cysts^1^** | 1 | Omitted |  |
|  | 2 | Omitted |  |
| **Sclerosis** | 1 | 1.34 (0.49, 3.67) | 0.571 |
|  | 2 | 1.37 (0.48, 3.94) | 0.561 |
| **Chondrocalcinosis** | 1 | 2.88 (1.15, 7.23) | 0.024 |
|  | 2 | 3.21 (1.28, 8.04) | 0.013 |

**GEE regression analysis of radiographic hip OA variables in HBM cases aged ≥65 years *vs.* Hertfordshire cohort study controls.** N (total no. of hip joints analysed)= 216 (HBM cases), 339 (HCS controls) except where indicated. Model 1=unadjusted, model 2=adjusted for age, gender and BMI. GEE = Generalised estimating equations with logistic link function. ^1^No cysts observed in cases therefore not analysed.

**Supplementary table 5**

| **Outcome** | **Model** | **OR (95% CI) in HBM cases *vs.* controls** | **p** |
| --- | --- | --- | --- |
| **Croft score ≥3** | 1 | 1.69 (0.99, 2.89) | 0.053 |
|  | 2 | 1.70 (0.99, 2.92) | 0.053 |
| **Osteophyte (any)** | 1 | 1.74 (0.93, 3.24) | 0.082 |
|  | 2 | 1.84 (0.96, 3.53) | 0.066 |
| **Moderate (≥grade 2) osteophyte** | 1 | 2.73 (1.48, 5.04) | 0.001 |
|  | 2 | 2.95 (1.57, 5.55) | 0.001 |
| **Any femoral osteophyte** | 1 | 1.64 (0.95, 2.83) | 0.074 |
|  | 2 | 1.70 (0.99, 2.91) | 0.053 |
| **JSN (any)** | 1 | 1.21 (0.73, 2.00) | 0.467 |
|  | 2 | 1.16 (0.70, 1.95) | 0.564 |
| **Moderate (≥grade 2) JSN** | 1 | 2.34 (0.90, 6.09) | 0.081 |
|  | 2 | 2.21 (0.88, 5.55) | 0.092 |
| **Cysts** | 1 | 1.00 (0.09, 11.1) | 0.997 |
|  | 2 | 0.75 (0.06, 8.71) | 0.817 |
| **Sclerosis** | 1 | 4.24 (1.54, 11.7) | 0.005 |
|  | 2 | 4.20 (1.56, 11.3) | 0.005 |
| **Chondrocalcinosis** | 1 | 2.48 (0.83, 7.45) | 0.106 |
|  | 2 | 2.64 (0.89, 7.85) | 0.081 |

**GEE regression analysis of radiographic hip OA variables in all male HBM cases *vs.* all male controls.** N (total no. of hip joints analysed) = 134 (HBM cases), 269 (controls) except where indicated. Model 1 = unadjusted, model 2 = adjusted for age and BMI. GEE = Generalised estimating equations with logistic link function.

**Supplementary table 6**

| **Outcome** | **Model** | **OR (95% CI) in HBM cases *vs.* controls** | **p** |
| --- | --- | --- | --- |
| **Croft score ≥3** | 1 | 1.59 (1.19, 2.13) | 0.002 |
|  | 2 | 1.58 (1.15, 2.17) | 0.005 |

**GEE regression analysis of radiographic hip OA in HBM cases *vs.* all controls, including hip replacements.** All total hip replacements classified as Croft score ≥3 (*ie.* assumed due to OA). Model 1 = unadjusted, model 2 = adjusted for age, gender and BMI. GEE = Generalised estimating equations with logistic link function. N (total no. of hip joints analysed) = 546 (HBM cases), 1737 (controls).

**Supplementary table 7**

| **Outcome** | **Model** | **OR (95% CI) in HBM cases *vs.* controls** | **p** |
| --- | --- | --- | --- |
| **Croft score ≥3** | 1 | 1.15 (0.71, 1.86) | 0.568 |
|  | 2 | 1.23 (0.70, 2.16) | 0.468 |
| **Osteophyte (any)** | 1 | 1.47 (1.00, 2.17) | 0.049 |
|  | 2 | 1.64 (1.07, 2.51) | 0.023 |
| **Moderate (≥grade 2) osteophyte** | 1 | 2.12 (1.25, 3.61) | 0.005 |
|  | 2 | 2.01 (1.10, 3.65) | 0.022 |
| **Any femoral osteophyte** | 1 | 1.66 (1.00, 2.77) | 0.049 |
|  | 2 | 1.99 (1.13, 3.49) | 0.017 |
| **JSN (any)** | 1 | 1.05 (0.67, 1.64) | 0.846 |
|  | 2 | 1.05 (0.63, 1.76) | 0.845 |
| **Moderate (≥grade 2) JSN** | 1 | 1.38 (0.58, 3.27) | 0.463 |
|  | 2 | 1.46 (0.54, 3.91) | 0.455 |
| **Cysts** | 1 | 0.27 (0.02, 3.00) | 0.287 |
|  | 2 | 0.28 (0.01, 7.93) | 0.452 |
| **Sclerosis** | 1 | 2.36 (0.83, 6.75) | 0.109 |
|  | 2 | 3.31 (0.91, 11.98) | 0.069 |
| **Chondrocalcinosis** | 1 | 1.67 (0.61, 4.58) | 0.315 |
|  | 2 | 1.81 (0.61, 5.32) | 0.282 |
|  | **Model** | **Mean difference (95% CI)** | **p value** |
| **Measured JSW**^1^ | 1 | 0.01 (-0.13, 0.14) | 0.938 |
|  | 2 | 0.11 (-0.03, 0.25) | 0.118 |

**GEE regression analysis of radiographic hip OA variables in HBM cases *vs.* family controls, excluding individuals with self-reported inflammatory arthritis.** Model 1 = unadjusted, model 2 = adjusted for age, gender and BMI. GEE = Generalised estimating equations with logistic / identity link function. N (total no. of hip joints analysed) = 495 (HBM cases), 270 (family controls) except where indicated. ^1^N=491 (HBM cases), 268 (family controls).

**Supplementary table 8**

| **Outcome (OA definition)** | **Model** | **OR (95% CI) in HBM cases *vs.* controls** | **p** |
| --- | --- | --- | --- |
| **Croft score ≥3** | 1 | 1.58 (1.17, 2.14) | 0.003 |
|  | 2 | 1.52 (1.09, 2.11) | 0.013 |
| **Croft score ≥3, requiring JSN^1^** | 1 | 1.51 (1.11, 2.05) | 0.008 |
|  | 2 | 1.42 (1.02, 1.98) | 0.038 |
| **Croft score ≥2** | 1 | 1.13 (0.86, 1.50) | 0.382 |
|  | 2 | 1.04 (0.76, 1.41) | 0.802 |
| **Croft score ≥4** | 1 | 2.10 (1.08, 4.07) | 0.028 |
|  | 2 | 1.99 (1.04, 3.81) | 0.037 |

**Effect of different hip OA definitions on HBM-hip OA association.** Model 1 = unadjusted, model 2 = adjusted for age, gender and BMI. Analyses used generalised estimating equations with logistic link function. N (total no. hip joints analysed) =530 (HBM cases), 1702 (combined controls). ^1^OA defined as JSN (≥grade 1) plus any other OA feature (osteophytes, subchondral sclerosis, cysts).

| **HBM control hips** | **N** | **Mean Z score (SD)** | **ttest p value for comparison** |
| --- | --- | --- | --- |
| **OA** | 29 | 0.56 (0.82) | 0.977 |
| **No OA** | 128 | 0.55 (0.87) |  |
| **HBM case hips** | **N** | **Mean Z score (SD)** | **ttest p value for comparison** |
| **OA** | 63 | 3.00 (1.10) | 0.807 |
| **No OA** | 256 | 2.96 (1.24) |  |

**Supplementary table 9**

**Comparison of total hip Z-score in hips with and without OA (Croft score >=3), stratified by HBM case status.** HBM study participants (cases and family controls) only. As most participants had either right or left hip DXA available (but not both), total hip Z-scores were available in a total of 476 hips (comprising 319 HBM and 157 family control hips), in 388 individuals (256 HBM cases, 132 family controls).
